# Antioxidant Properties of Thymoquinone, Thymohydroquinone and Black Cumin (*Nigella sativa* L.) Seed Oil: Scavenging of Superoxide Radical Studied Using Cyclic Voltammetry, DFT and Single Crystal X-ray Diffraction

**DOI:** 10.3390/antiox12030607

**Published:** 2023-03-01

**Authors:** Raiyan Sakib, Francesco Caruso, Sandjida Aktar, Stuart Belli, Sarjit Kaur, Melissa Hernandez, Miriam Rossi

**Affiliations:** Department of Chemistry, Vassar College, Poughkeepsie, NY 12604, USA

**Keywords:** thymoquinone, thymohydroquinone, black seed oil, cyclovoltammetry, superoxide, DFT

## Abstract

Black cumin seeds and seed oil have long been used in traditional foods and medicine in South Asian, Middle Eastern and Mediterranean countries and are valuable flavor ingredients. An important ingredient of black cumin is the small molecule thymoquinone (TQ), which manifests low toxicity and potential therapeutic activity against a wide number of diseases including diabetes, cancer and neurodegenerative disorders. In this study, the antioxidant activities of black seed oil, TQ and a related molecule found in black cumin, thymohydroquinone (THQ), were measured using a direct electrochemical method to experimentally evaluate their superoxide scavenging action. TQ and the black seed oil showed good superoxide scavenging ability, while THQ did not. Density Functional Theory (DFT) computational methods were applied to arrive at a chemical mechanism describing these results, and confirmed the experimental Rotating Ring Disk Electrode (RRDE) findings that superoxide oxidation to O_2_ by TQ is feasible, in contrast with THQ, which does not scavenge superoxide. Additionally, a thorough inquiry into the unusual cyclic voltammetry pattern exhibited by TQ was studied and was associated with formation of a 1:1 TQ-superoxide radical species, [TQ-O_2_]^−^•. DFT calculations reveal this radical species to be involved in the π-π mechanism describing TQ reactivity with superoxide. The crystal structures of TQ and THQ were analyzed, and the experimental data reveal the presence of stacking intermolecular interactions that can be associated with formation of the radical species, [TQ-O_2_]^−^•. All three of these methods were essential for us to arrive at a chemical mechanism that explains TQ antioxidant activity, that incorporates intermolecular features found in the crystal structure and which correlates with the measured superoxide scavenging activity.

## 1. Introduction

The annual herb *Nigella sativa* L. is native to the Mediterranean region, northern Africa and south Asia. Historical evidence of its use by ancient civilizations in those areas exists and seeds of the plant (black cumin) were even found in King Tutankhamen’s tomb [1]. Today, populations in those geographical locations still use the black seeds and the related oil, containing the phytochemicals thymoquinone (TQ) and thymohydroquinone (THQ), in traditional foods as valuable flavor ingredients and for medicinal purposes, ingested with food or honey [1,2]. The main active phytochemicals in the seeds and oil include several monoterpenes, TQ (by far, the highest concentration), THQ, p-cymene, carvacrol and thymol [3]. Additionally, black cumin seed oil is rich in nutritionally beneficial fatty acids such as linoleic acid (57.71%) and oleic acid (24.46%) [2]. As with many agricultural materials, black seeds and the related cold-pressed oil show compositional variation depending on the area of cultivation [4].

Importantly, black seeds and their oil have demonstrated widespread action against numerous diseases. Because the low toxicity and potential therapeutic activity of TQ is notable, including diseases such as diabetes, cancer and neurodegenerative disorders, it has been the subject of many review articles [5,6,7]. Recently, TQ therapeutic activity has extended to include its use as a viable treatment against Covid-19 [8]. The presence of TQ and THQ in other plant species besides *N. Sativa*, including the *Thymus vulgaris* L. and *Origanum* species, is confirmed [5,9]. Studies on the biosynthesis of TQ and THQ in Lamiaceae established that the monoterpenes, thymol and carvacrol, are used to produce THQ by cytochrome P-450 enzymes, CYP76S40 or CYP736A300, which then is converted to TQ [10].

An objective of this study was to obtain chemical data to help clarify the relationship between the antioxidant and free radical scavenging properties of these substances and to gain insight into the chemical mechanisms underlying the therapeutic benefits of these phytochemical ingredients. We investigated the crystal and molecular structures of the small molecules TQ and THQ, as this method also reveals information regarding intermolecular interactions among neighboring molecules. We measured the antioxidant activity of black seed oil, TQ and THQ using the RRDE and one-electrode cyclic voltammetry electrochemical methods to obtain information about scavenging properties. Last, we used DFT methods to describe electronic properties since these are related to a molecule’s ability to donate and/or receive electron density and its antioxidant capability. These computational methods were essential for us to arrive at a superoxide scavenging chemical mechanism that incorporates intermolecular features found in the crystal structure and which correlates with the measured superoxide scavenging activity.

## 2. Materials and Methods

### 2.1. Reagents

Thymoquinone (Toronto Research Chemicals, Toronto, ON, CA), thymohydroquinone (Cayman Chemical, Ann Arbor, MI, USA) and Cold Pressed Black Seed Oil (Maju Superfoods, San Diego, CA, USA). For electrochemical studies, tetrabutylammonium bromide (TBAB; TCI Chemicals, Portland, OR, USA) and 99.9% anhydrous Dimethyl Sulfoxide (DMSO; Sigma-Aldrich, Inc., St. Louis, MO, USA).

### 2.2. Equipment

#### 2.2.1. Electrochemistry

A Pine Research WaveDriver 20 bipotentiostat with the Modulated Speed Electrode Rotator was used to perform the hydrodynamic voltammetry at a rotating ring-disk electrode (RRDE) as well as cyclic voltammetry (CV). The working electrode is the AFE6R2 gold disk and gold ring rotator tip (Pine Research, Durham, NC, USA) combined with a coiled platinum wire counter electrode and a reference electrode consisting of an AgCl coated silver wire immersed in 0.1 M tetrabutylammonium bromide (TBAB) in dry DMSO in a fritted glass tube. The electrodes were placed in a five-neck electrochemical cell together with means for either bubbling or blanketing the solution with gas. Voltammograms were collected using Aftermath software provided by Pine Research. Careful cleaning of the electrodes was performed by polishing with 0.05 µm alumina-particle suspension (Allied High Tech Products, Inc., Rancho Dominguez, CA, USA) on a moistened polishing microcloth to eliminate potential film formation [11]. Cyclic voltammetry was performed using the same electrodes and cell.

#### 2.2.2. X-ray Diffraction

An APEX2 DUO platform X-ray diffractometer from Bruker Advanced X-ray Solutions (Madison, WI, USA) was used to obtain X-ray data measurements on suitable crystals at 125 K. Temperature was maintained using a cold liquid nitrogen stream from Oxford Cryosystems (UK).

### 2.3. Electrochemical Studies

#### 2.3.1. Hydrodynamic Voltammetry (RRDE)

Stock clear light-yellow 0.03 M solution of TQ (0.0492 g in 10 mL) and clear colorless 0.03 M solution THQ (0.0498 g in 10 mL), in anhydrous DMSO (99.9% purity), were used in trials, whereas black seed oil was directly introduced in the electrochemical cell.

For the experiment, a solution of 0.1 M TBAB electrolyte in anhydrous DMSO was bubbled for 5 min with a dry O_2_/N_2_ (35%/65%) gas mixture to establish the dissolved oxygen level in the electrochemical cell of 50 mL. The Au/Au disc electrode was then rotated at 1000 rpm and potential sweep applied to the disk from 0.2 V to −1.2 V and then back to 0.2 V while the ring was held constant at 0.0 V; the disk voltage sweep rate was set to 25 mV/s. The molecular oxygen reduction peak (reaction 1) is observed around −0.6 V, at the disk electrode; the oxidation current (reaction 2) occurs at the ring electrode. An initial blank, in the absence of an antioxidant, was run on this solution and the ratio of the ring/disk current was calculated as the “efficiency”. This blank efficiency was found to be about 20%. Next, an antioxidant aliquot was added, the solution bubbled with the gas mixture for 5 min, the voltammogram was rerecorded, and efficiency obtained. In this way, the rate at which increasing concentrations of antioxidant scavenge the generated superoxide radicals during the electrochemical reaction is determined as each additional antioxidant aliquot is added. Results from each run were collected on Aftermath software and represented as voltammograms showing current vs. potential graphs that were later analyzed using Microsoft Excel. The aliquots used are indicated in related RRDE graphs. Ultimately, the slope of the overall decrease in efficiency with addition of antioxidant serves as a quantitative measure of the antioxidant activity of each compound. Any decrease in the collection efficiency is expected to be due to the amount of superoxide removed by the antioxidant.

In an RRDE voltammetry experiment, the generation of the superoxide radicals occurs at the disk electrode while the oxidation of the residual superoxide radicals (that have not been scavenged by the antioxidant) occurs at the ring electrode.

Reaction 1: Reduction of molecular oxygen at the disk electrode

Disk Reaction            O_2_ + e^−^ → O_2_•^−^ (1)

Reverse Reaction 2: Oxidation of superoxide radicals at the ring electrode

Ring Reaction            O_2_•^−^ → O_2_ + e^−^ (2)

#### 2.3.2. Cyclic Voltammetry

On completion of the RRDE study of TQ, CV was performed both in the presence and absence of oxygen dissolved in the DMSO solvent in order to further study the reactions occurring at the electrode surfaces.

### 2.4. Computational Study

Calculations were performed using software programs from Biovia (San Diego, CA, USA). Density Functional Theory (DFT) included in DMol^3^ was applied to calculate energy, geometry, and frequencies implemented in Materials Studio 7.0 (PC platform) [12]. We employed the double numerical polarized (DNP) basis set that included all the occupied atomic orbitals plus a second set of valence atomic orbitals, and polarized d-valence orbitals [13]; the correlation generalized gradient approximation (GGA) was applied including Becke exchange [14] plus BLYP-D correlation including Grimme’s correction when van der Waals interactions were involved [15]. All electrons were treated explicitly and the real space cutoff of 5 Å was imposed for numerical integration of the Hamiltonian matrix elements. The self-consistent field convergence criterion was set to the root mean square change in the electronic density to be less than 10^−6^ electron/Å^3^. The convergence criteria applied during geometry optimization were 2.72 × 10^−4^ eV for energy and 0.054 eV/Å for force. Calculations included the effect of DMSO solvent using the continuous model of Dmol^3^ [16], to allow correlation with the experimental features from cyclovoltammetry results.

### 2.5. Diffraction Study

Suitable large yellow single crystals of TQ were grown from a 2:1 (*v*/*v*) methanol:water solution. Colorless crystals of THQ were recrystallized from 1:1 (*v*/*v*) ethanol:water solution. The crystal structures were solved and refined using full-matrix least-squares on F^2^ with the Bruker incorporated ShelX programs [17]. We input the X-ray data into the MERCURY program from Cambridge Structural Database (CSD) to produce images of the molecules and crystal packing [18]. Crystal data of THQ and TQ have been deposited at the CSD and are available at https://www.ccdc.cam.ac.uk/structures/? (accessed on 1 January 2023) using Identifier CCDC number 2239493-2239494 and are available upon request [19].

## 3. Results and Discussion

### 3.1. Diffraction Study

**Figure 1:**
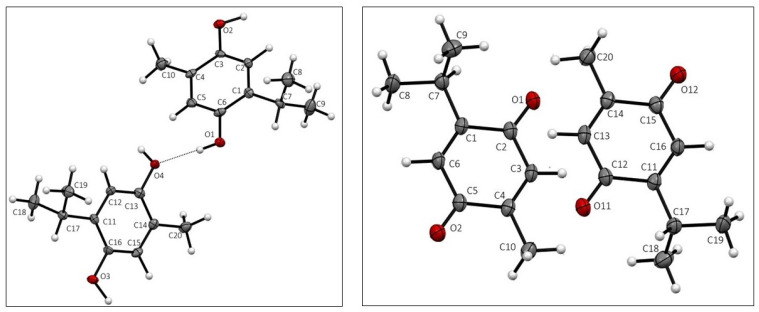
Asymmetric unit of THQ, (**left**), and TQ, (**right**), with atom labeling.

Crystal data for THQ and TQ are given in Table 1. Both THQ and TQ crystallized with two molecules in the asymmetric unit, displayed in Figure 1. Atomic distances and angles in both structures agree with expected values.

#### 3.1.1. Thymoquinone

The two molecules in the asymmetric unit differ mainly in the rotation of the isopropyl group, as seen in Figure S1.

**Figure 2:**
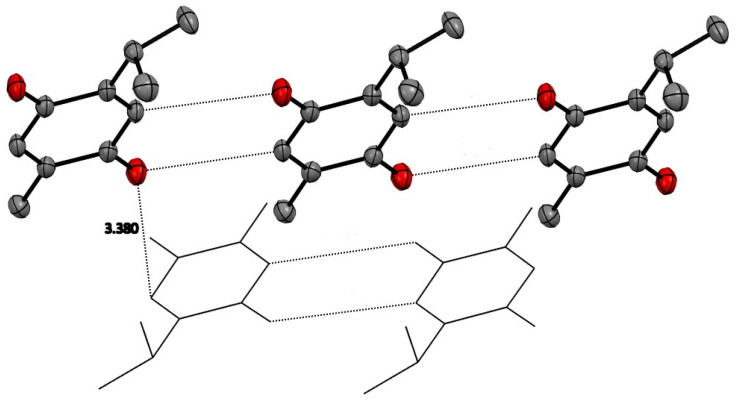
TQ molecules have planar quinone rings which have close contacts with other TQ molecules in the same plane of about 3.4 Å. Stacking between these planes is also about 3.4 Å. Hydrogen atoms removed for clarity.

Alternating inversion center related molecules of TQ are stacked in a displaced manner primarily along the *a* axis, allowing the hydrophobic isopropyl and methyl groups to be close to each other. The displaced stacking among these planes is close to 3.4 Å. Similarly, TQ molecules in the same plane are arranged with CH…O close contacts of about 3.4 Å. These are displayed in Figure 2 and illustrate that only van der Waals forces are seen in the crystal structure.

A lower-resolution powder diffraction structure of thymoquinone had been earlier reported with CSD refcode NIDKER [20]. This structure determination was not able to detect the hydrogen atoms in the molecule.

#### 3.1.2. Thymohydroquinone

**Figure 3:**
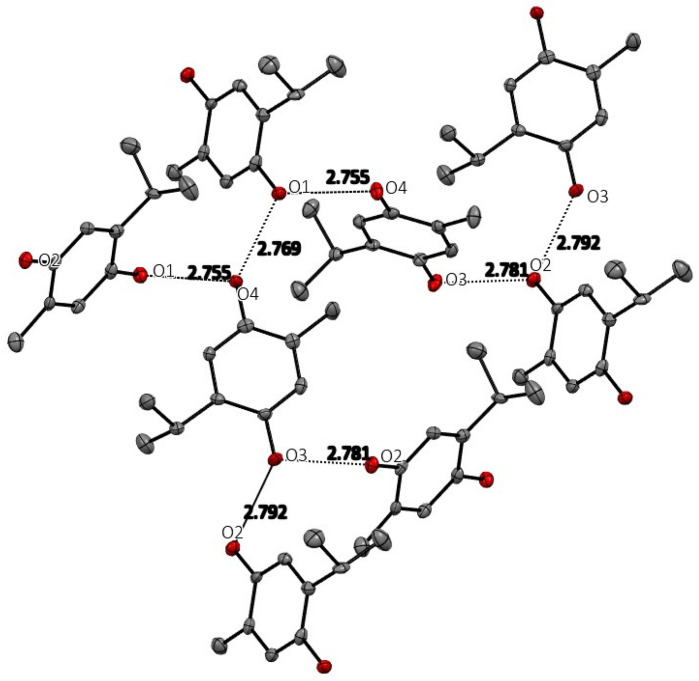
Hydrogen bonding among the THQ molecules in the crystal. Hydrogen atoms are not shown for clarity.

The space group for THQ was ultimately chosen to be the non-centric space group Pc. The centrosymmetric P2_1_/c was attempted, but refinement in that space group gave an unacceptable solution with much higher R-value and abnormally high anisotropic displacement parameters. As expected, THQ manifests extensive and strong hydrogen-bonding intermolecular interactions, which are shown in Figure 3, and whose geometric details are reported in Table 2. Each hydroxyl group (O1H, O2H in Molecule 1 and O3H and O4H of Molecule 2 in the asymmetric unit) has two strong intermolecular hydrogen bonds creating an infinite hydrogen bond network throughout the crystal structure. No stacking interactions are evident.

### 3.2. DFT Study

#### 3.2.1. Thymoquinone

**Figure 4:**
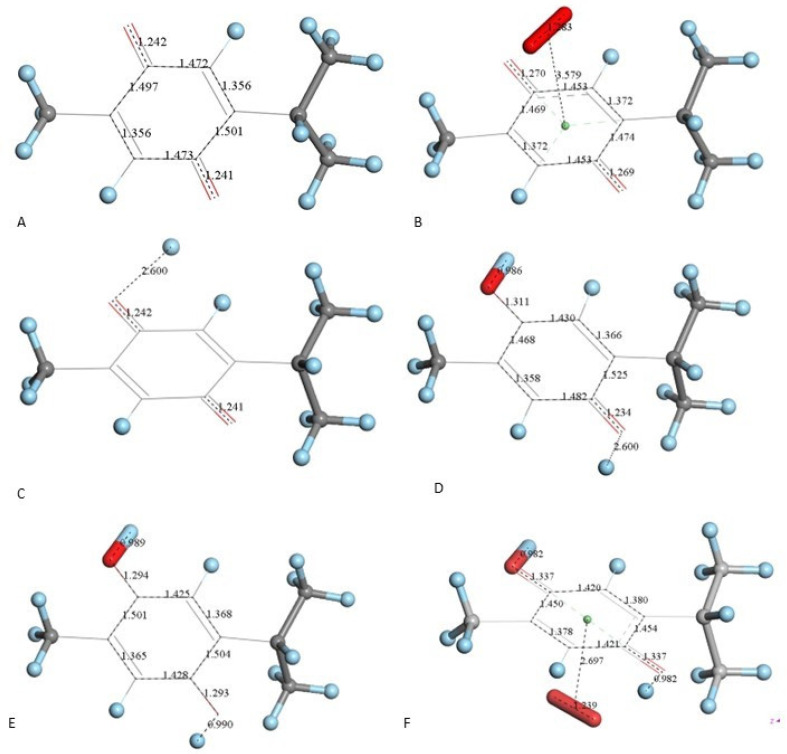
TQ superoxide scavenging DFT calculations. H atoms are light blue to avoid white background misperception. (**A**) Minimized TQ molecule obtained after input of corresponding X-ray coordinates. (**B**) Superoxide was π-π placed (3.50 Å) above ring shown in A, and upon DFT minimization both moieties became more distant, 3.579 Å, while ring C-C bond lengths were modified due to electron transferred from superoxide to the ring and O-O bond length shortens to 1.283 Å. (**C**) Structure shown in A has a proton van der Waals separated (2.60 Å) from one O(carbonyl). (**D**) DFT outcome of structure shown in C, plus an additional proton 2.60 Å separated from the second O(carbonyl). (**E**) DFT outcome of structure shown in D whose charge is 2+, showing two C-OH moieties. (**F**) A superoxide radical was π-π placed in E, and DFT shows formation of the radical [thymohydroquinone-η-O_2_]•^+^, with separation between centroids of 2.697 Å.

After X-ray atomic coordinates of TQ were input in Materials Studio (Dmol^3^) quantum mechanical program, geometry optimization was applied and the resulting minimum energy structure is shown in Figure 4A. Single bonds in the quinone ring are in the range 1.47–21.501 Å, obviously longer than the two double bonds, 1.356 Å (compared with X-ray values of 1.473–1.493 Å (single) and 1.337–1.342 Å (double)).

Next, we studied the TQ structural modifications after a superoxide radical was π-π placed at van der Waals separation (3.50 Å) between quinone and superoxide centroids. DFT geometry minimization, Figure 4B, shows the superoxide being more distant, 3.579 Å apart, and with the initial superoxide O-O bond length distance of 1.373 Å shortening to 1.283 Å, which rather corresponds to the O_2_ double bond distance. Meanwhile, the TQ ring bond lengths become modified, for instance, C=C double bonds become longer, 1.372 Å (from initial values shown in Figure 4A 1.356 Å), while the quinone single C-C bonds become 0.02–0.03 Å shorter. Shorter single bonds and longer double bonds can be associated with ring aromatization, suggesting the unpaired electron of superoxide is donated to the quinone ring. This result has already been observed by us in related polyphenols scavenging superoxide [21]. The electron captured by the ring also has an effect on the C=O bond lengths as they become elongated by 0.03 Å. We call this process (a). From Figure 4 we conclude that superoxide oxidation (and O_2_ formation) by TQ is feasible.

**Figure 5:**
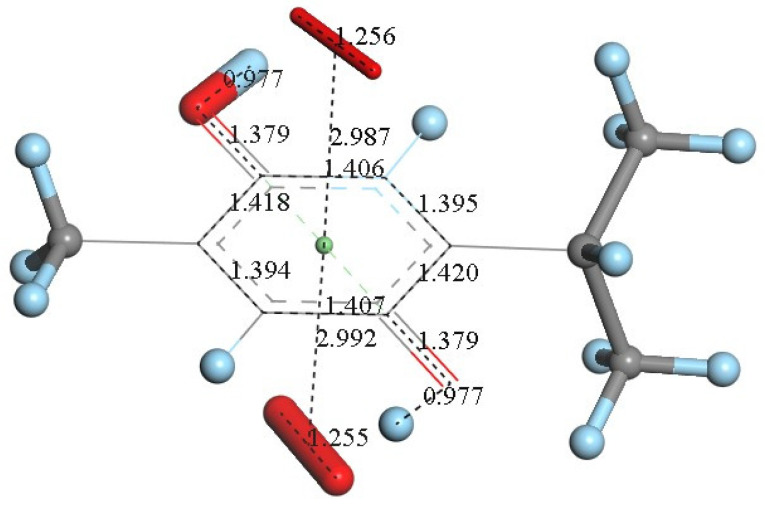
An additional superoxide (large stick style) was π-π placed below the structure shown in Figure 4F and its DFT minimization shows the neutral final complex [thymohydroquinone-η-O_2_], obtained for process (b).

However, since TQ has no hydroxyls available, an alternative process (b) can be envisioned involving interaction between the quinone and available protons. This route can induce reduction of a C=O carbonyl to a C-OH moiety and has been described for the quinone natural product embelin [22]. The initial state of this process is shown in Figure 4C, where a proton is placed at van der Waals separation from the O(carbonyl), 2.60 Å (1.20 Å + 1.40 Å, for H and O van der Waals radii, respectively), Figure 4A. After DFT minimization, Figure 4D shows the proton captured by the TQ, O-H bond length = 0.986 Å, and lengthening of the corresponding C-O bond, 1.311 Å, compared to initial 1.241 Å in Figure 4A, thus confirming C-OH formation. Indeed, Figure 4D shows the input for the next DFT calculation, i.e., the approach of an additional proton to the second O(carbonyl) in TQ, located initially at 2.60 Å, and which results in the energy minimum moiety shown in Figure 4E, a 2+ charged polyphenol (from 2 captured protons), and having 2 hydroxyls, [THQ]^2+^. As observed in Figure 4B, superoxide is able to transfer its unpaired electron to the TQ ring, and we can expect that the dicationic polyphenol in Figure 4E will permit an even easier electron transfer to the ring. Indeed, this happens and the DFT outcome after energy minimization of the initial structure, a superoxide π-π posed at 3.50 Å (Figure 4E), is shown in Figure 4F, having shorter separation between stacked centroids, 2.697 Å. Upon an additional π-π attack by a second superoxide on the opposite side of the polyphenol ring, this radical also binds to the ring with a longer separation, 2.987 Å, Figure 5. This resulting thymohydroquinone-η-2O_2_ complex is a neutral species with aromatic character, as shown by the ring C-C bonds in the range 1.394–1.418 Å; this is process (b). Attempts to extract one H(hydroxyl) from the thymohydroquinone-η-2O_2_ structure shown in Figure 5, by an additional superoxide via σ attack, were not successful, as the possible expected radical product {[thymohydrosemiquinone-η-2O_2_]• HO_2_^−^} is +4.3 Kcal/mol higher than the reagent, thus indicating no further reactivity of the thymohydroquinone-η-2O_2_ complex.

As recently described, molecular oxygen is an important product when polyphenols act as mimics of superoxide dismutase enzymes, following reaction (3) [23].

2 O_2_•^−^ + 2H^+^ → O_2_ + H_2_O_2_ (3)

The reacting proton component of reaction (3) is associated with a polyphenol hydroxyl H atom transfer (HAT, σ attack, [23]) and is more often described in polyphenol antioxidant studies than the π-π attack. According to our DFT results, in TQ scavenging of superoxide, the reaction (3) reactants are involved, since two protons (Figure 4C,D) are captured as well as two superoxide radicals (Figure 4F and Figure 5), but the products of reaction (3), O_2_ and H_2_O_2_, are not produced by process (b). However, the independent process (a), shown in Figure 4B, suggests O_2_ formation. In any case, H_2_O_2_ is not produced, and so TQ does not mimic SOD action, unlike behavior recently described for some polyphenols [23]. We conclude that TQ scavenging of superoxide can be described only in terms of stoichiometric reactions.

#### 3.2.2. Thymohydroquinone

**Figure 6:**
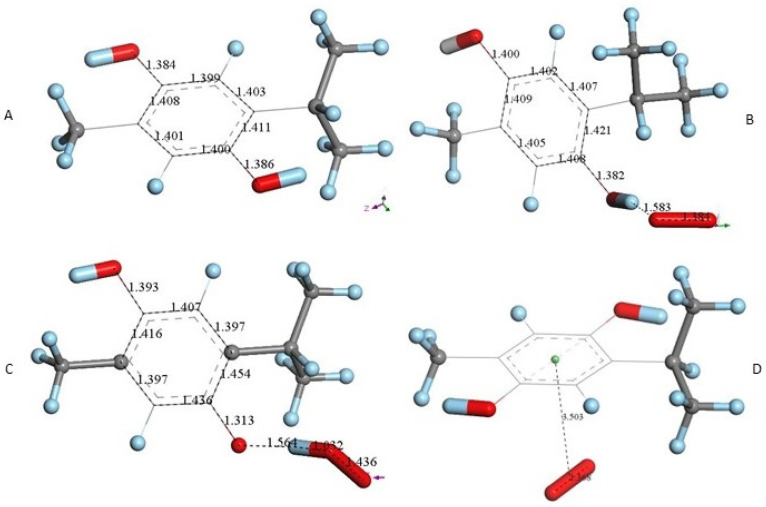
THQ DFT calculations. (**A**) Geometry minimization of thymohydroquinone X-ray coordinates. (**B**) Minimization obtained after placing a van der Waals separated superoxide from one H(hydroxyl). (**C**) Minimization obtained after placing a van der Waals separated HO_2_^−^ from thymohydroquinone radical. (**D**) Minimization obtained after placing a π-π van der Waals separated superoxide from THQ, 3.50 Å. Initial superoxide bond length of 1.373 Å had only a slight variation, 1.368 Å. This indicates rejection between both reagents.

THQ X-ray atomic coordinates were treated in a similar way with Dmol^3^. The DFT energy minimum structure Figure 6A shows aromatic ring distances (1.40–1.41 Å), which can be compared to the corresponding X-ray values (1.37–1.41 Å). A superoxide σ-oriented towards one H(hydroxyl), initially at van der Waals separation 2.60 Å, results in a structure having shorter separation, 1.583 Å, Figure 6B. This structure was compared with the one obtained after posing a HO_2_**^−^** anion near the corresponding thymohydrosemiquinone radical (a calculation describing the potential expected product), which resulted in a slightly shorter H–O separation, 1.564 Å, Figure 6C. A comparison between the results of both minimizations showed ΔG of 0.3 kcal/mol, indicating no product feasibility. The π-π attack was also analyzed after placing superoxide on top of Figure 6A, and DFT minimization showed both reagents rejected, Figure 4D. We conclude that THQ does not scavenge superoxide.

### 3.3. Electrochemistry

#### 3.3.1. Thymoquinone

**Figure 7:**
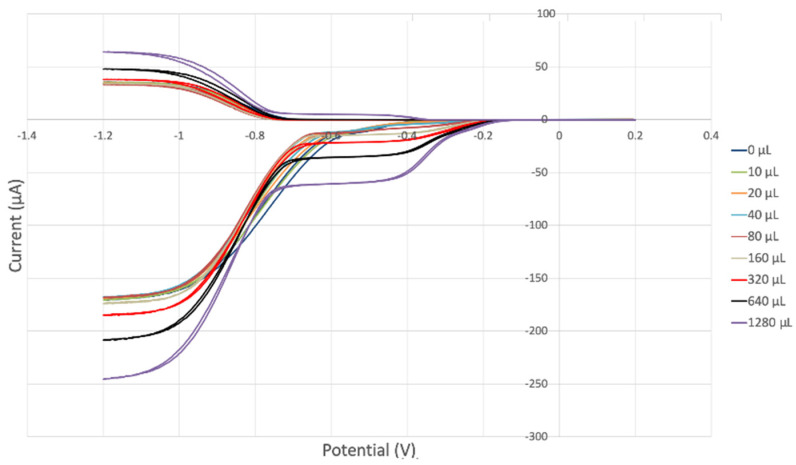
TQ RRDE cyclovoltammetry.

**Figure 8:**
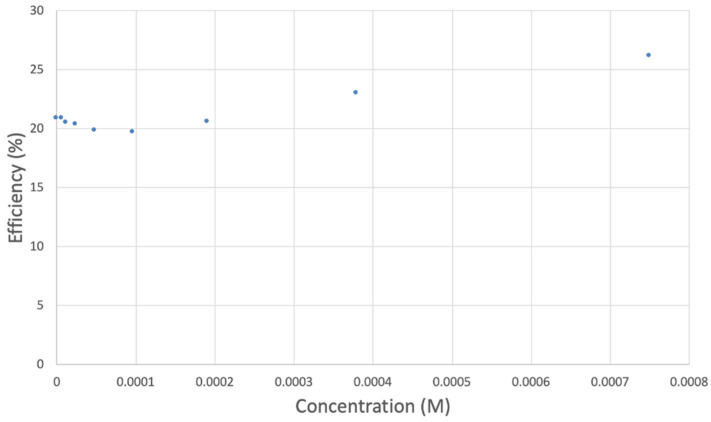
TQ efficiency.

**Figure 9:**
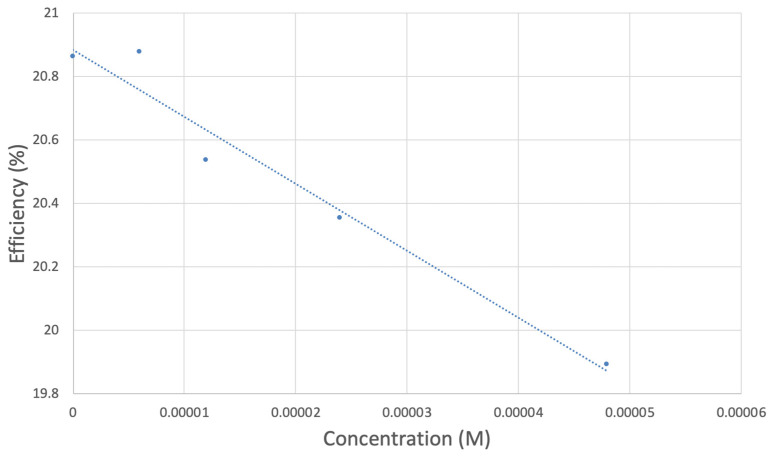
TQ collection efficiency for blank plus first four aliquots, whose line equation is y = −21104x + 20.9 (R^2^ = 0.9965).

**Figure 10:**
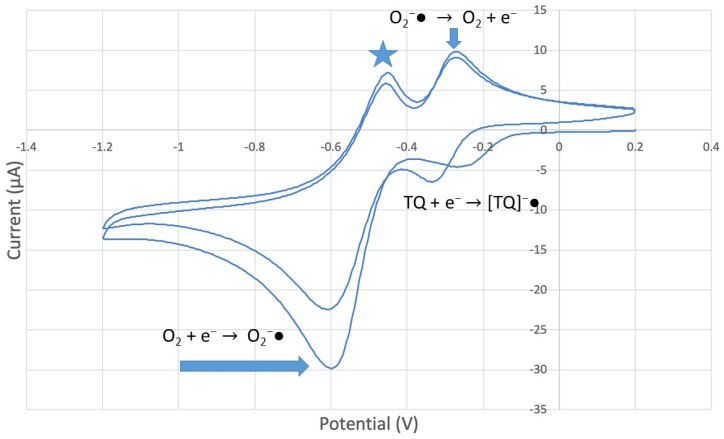
CV of TQ 0.00074 M solution (1280 µL aliquot) saturated with the O_2_/N_2_ mixture. The star and arrows are described in the text.

**Figure 11:**
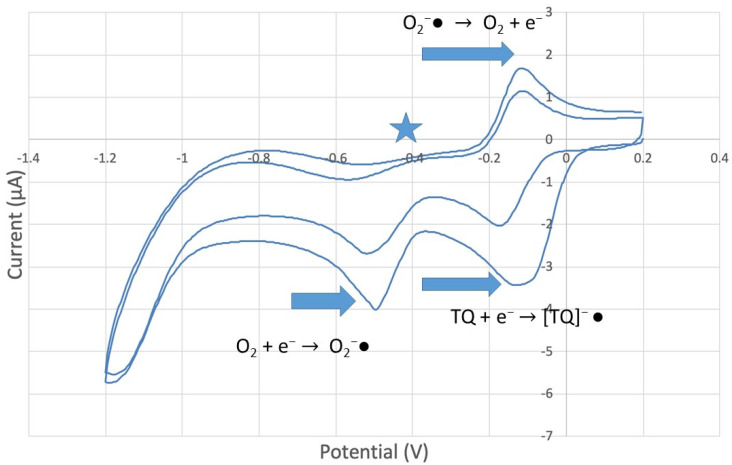
CV of TQ 0.00074 M solution (1280 µL aliquot) purged with N_2_ gas showing evidence of incomplete purging of the O_2_.

The antioxidant activity of TQ was studied using an electrochemical technique developed by Belli et al. [24]. Figure 7 shows RRDE graphs of TQ for all aliquots. Figure 8 shows the corresponding collection efficiency, while Figure 9 shows the collection efficiency of the initial five TQ additions. In addition, CV was performed on a O_2_ saturated solution (bubbled 5 min) containing a single TQ aliquot (Figure 10), and then after purging with dry N_2_ gas, Figure 11. Both CVs show two complete scans.

**Figure 12:**
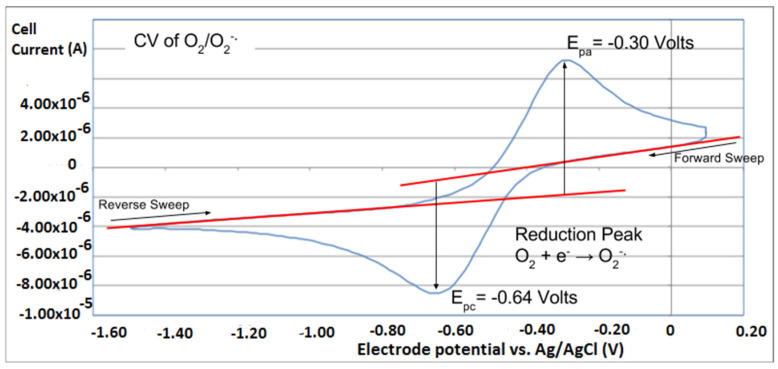
A cyclovoltammogram of a blank experiment using a classical one working electrode, is taken from [24], where meaning of red lines are described.

The collection efficiency for the TQ RRDE experiment (Figure 8) is anomalous, showing an initial decrease in efficiency through the first four TQ additions (as normally observed), but then the efficiency increased with each further addition of TQ aliquot, which has never been observed in previous studies [22,24,25,26,27]. To clarify this behavior of TQ, cyclic voltammetry of an RRDE solution yielded the voltammogram in Figure 10. Under these conditions, the reduction of oxygen to generate superoxide, reaction (1), −0.60 V, as well as the reverse reaction (2) (oxidation of superoxide), −0.30 V, was previously examined by analyzing a blank solution (without antioxidant), Figure 12 [24]. We analyzed the peaks seen in Figure 10, starting with the first reduction peak (non-reversible) at about −0.30 to −0.35 V and assigned it to incorporation of an electron into TQ (TQ + e- → [TQ]^●^). A reverse reaction was not observed, as after reversing the potential (more positive) than 0.20 V, no related oxidation peak was seen (not shown).

**Figure 13:**
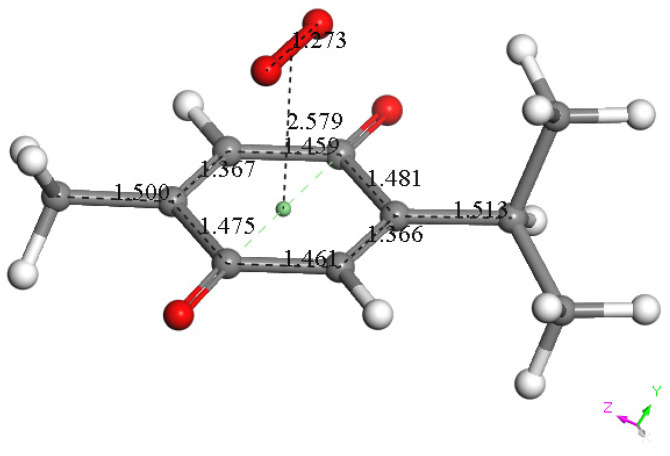
DFT minimum energy reaction product, obtained after geometry minimization of π-π approach of a van der Waals separated, 3.50 Å, molecular oxygen and the TQ radical [TQ]•^−^. This radical product had a shortened π-π interaction of 2.579 Å and is associated with 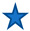 peak oxidation in Figure 9, to give TQ plus O_2_.

To assign the first oxidation peak, labelled 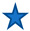 in Figure 10 at about −0.45 V, we used DFT to analyze the reactivity of [TQ]^−^• (seen at peak −0.30 to −0.35 V), and saw that this species is able to react with bubbled oxygen to form the radical [TQ-O_2_]^−^•, Figure 13, a reaction not influenced by the further negative electrochemical potential. This capture of O_2_ by a radical species is uncommon, and the resulting species, [TQ-O_2_]^−^•, is therefore associated with its oxidation 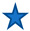 peak in Figure 10. By purging the electrochemical cell with bubbled nitrogen, in Figure 11, the 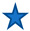 peak was strongly decreased, consistent with decreasing O_2_, although not completely eliminated because of the presence of some residual non-purged oxygen. Thus, the anomalous increase in efficiency, Figure 8, is not associated with antioxidant features of TQ, and contrary to results seen for previously studied scavengers [22,24,25,26,27]. Rather, this increase in efficiency is due to increasing [TQ-O_2_]^−^• concentration, formed as TQ aliquots are added and react with bubbled O_2_.

However, it is interesting to compare the peaks associated with reactions (1) and (2) in Figure 10, as reaction (2) peak height (indicated by short vertical arrow) is shorter than that of reaction (1) (represented by longer horizontal arrow). For comparison, Figure 12 shows a typical blank, also using one working electrode, where both peak heights are seen to be equal. Thus, in Figure 10, the difference in peak height between the amount of superoxide present at the oxidation peak and the amount of superoxide at the reduction peak is due to TQ scavenging of superoxide. These features have been characterized in a previous study using both electrochemical methods, RRDE and CV [24]. The interference of radical [TQ-O_2_]^−^• seems not to be important at low concentrations of TQ, as seen in the first five data points in Figure 8. These initial values are plotted in Figure 9 to obtain the efficiency at low concentrations; the related slope (slope = −2.11 × 10^4^) for the linear expression y = −21104x + 20.9 (R^2^ = 0.9965), is a good indicator of superoxide scavenging ability of TQ. We conclude that RRDE experiments on TQ show a mixed effect: on one hand, at low concentrations, TQ is able to scavenge superoxide, and shows the expected decreasing efficiency while at higher TQ concentrations, an unexpected increase in efficiency can be associated with formation of the [TQ-O_2_]^−^• radical. This radical species is oxidized after reversing the potential at about −0.45 V in the electrochemistry experiment using one working electrode, or alternatively, is detected at the ring electrode, whose setting is 0.0 V in the RRDE experiment.

Table 3 shows electrochemical RRDE data of other polyphenols analyzed previously. The slope of TQ, −2.11 × 10^4^ falls between chrysin and eriodyctiol, but is closer in value to the latter.

Our results support earlier investigations that describe TQ superoxide scavenging ability [28] as well as a thorough inquiry on the unusual cyclic voltammetry pattern shown by TQ [29].

#### 3.3.2. Thymohydroquinone

**Figure 14:**
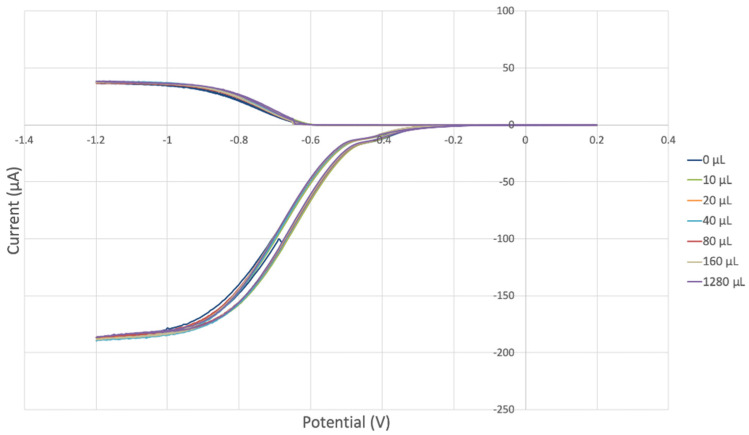
THQ is not able to scavenge superoxide, as no variation is found with increasing concentration of THQ.

THQ was also explored with the RRDE method, and results are shown in Figure 14. There was no variation in disk and ring current, and so THQ does not scavenge superoxide, in agreement with the results of the DFT study described earlier, and so the data show no change from the blank run, both below and above the potential axis, indicating unmodified reaction (1) and (2), respectively. In other words, THQ has no effect on the RRDE outcome.

#### 3.3.3. Black Seed Oil

**Figure 15:**
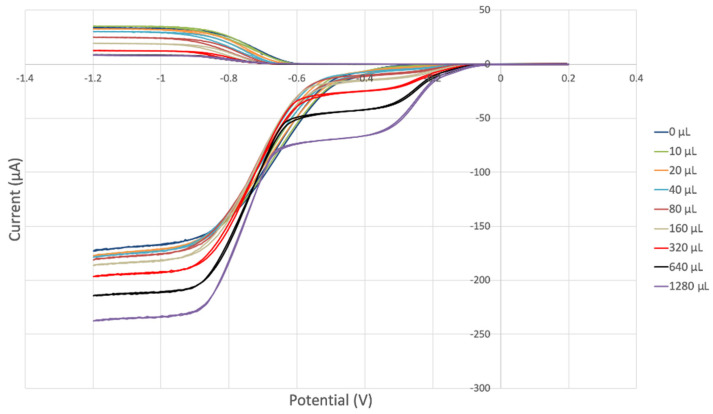
Black seed oil RRDE electrochemistry.

**Figure 16:**
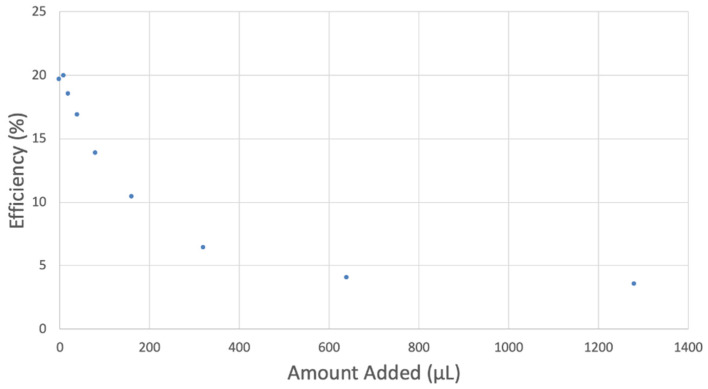
Black seed oil collection efficiency.

**Figure 17:**
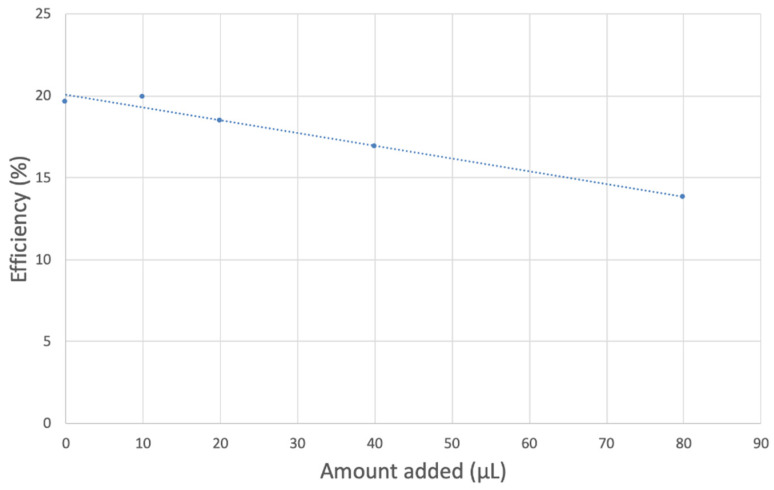
Black seed oil collection efficiency of blank plus first four aliquots, y = −0.0781x + 20.08, R^2^ = 0.9763.

Black seed oil was also analyzed, and its RRDE cyclovoltammetry data are shown in Figure 15. Figure 16 shows decreasing collection efficiency overall, with the greatest decrease at low concentrations. Figure 17 shows the collection efficiency of the first four aliquots, having good linear behavior (y = −0.0781x + 20.08, R^2^ = 0.9763), demonstrating a good scavenging ability by the black seed oil.

Earlier RRDE studies by our group on another cold-pressed vegetable oil, extra virgin olive oil [30], allow us to compare the slope of the RRDE efficiency of black seed oil (−0.078) and that of extra virgin olive oil (−0.0838). The latter is characterized by a linear trend (y = −0.0838x +19.73, R^2^ = 0.99348) for all 11 runs, (aliquot range 0–100 µL), and shows a slightly better scavenging of superoxide by olive oil.

## 4. Conclusions

The molecular mechanisms that underlie the therapeutic effects of TQ are not completely understood, and literature reports show that TQ likely interacts with a number of receptors. Its low toxicity and the fact that TQ as well as the oil and seeds from which it is isolated have been used for thousands of years, make studying its therapeutic capacity appealing [6].

In this work, we have measured the superoxide scavenging ability of cold-pressed black cumin oil and two of its ingredients, TQ and THQ. Our results show that the oil and TQ are strong scavengers of the superoxide radical, whereas THQ has no effect on the scavenging, despite the presence of two para-hydroxyl groups. Structural X-ray diffraction data support subsequent DFT calculations that show a path of energetically feasible molecular transformations which corroborate cyclovoltammetry experimental features, i.e., the scavenging of superoxide by TQ, and no scavenging by THQ. The suggested stoichiometric reactivity of TQ when reacting with superoxide involves 1) direct π-π attack, involving superoxide oxidation to produce O_2_ as a product, and 2) superoxide σ proton attack on a TQ O(carbonyl), which gets reduced to C-OH, and is not sensitive to further attack by superoxide.

This theoretical assessment is confirmed using two cyclovoltammetry techniques, cyclic voltammetry and hydrodynamic voltammetry at an RRDE. Lastly, our work shows the efficacy of combining multidisciplinary approaches, including experimental X-ray crystallography and voltammetry as well as computational DFT methods, to provide understanding of the mechanism of action of small molecule antioxidants. This research supports further exploration of other natural products having important biomedical activities.

## Figures and Tables

**Table 1:** Crystal data and structure refinement for thymohydroquinone (THQ), thymoquinone (TQ).

Identification code	THQ	TQ
Empirical formula	C_10_H_14_O_2_	C_10_H_12_O_2_
Formula weight	166.221	164.206
Temperature/K	125.15	125.15
Crystal system	monoclinic	triclinic
Space group	Pc	P-1
a/Å	10.7442(11)	6.6965(7)
b/Å	10.020(1)	10.4246(10)
c/Å	9.0363(9)	13.4314(13)
α/°	90	98.553(5)
β/°	110.201(2)	102.981(5)
γ/°	90	92.841(5)
Volume/Å^3^	912.98(16)	900.15(16)
Z	4	4
ρ_calc_ g/cm^3^	1.209	1.212
μ/mm^−1^	0.083	0.673
F(000)	360.2	353.2
Crystal size/mm^3^	0.1 × 0.08 × 0.06	0.29 × 0.25 × 0.01
Radiation	Mo Kα (λ = 0.71073)	Cu Kα (λ = 1.54178)
2Θ range for data collection/°	4.04 to 50.92	6.84 to 143.16
Index ranges	−12 ≤ h ≤ 12, −12 ≤ k ≤ 12, −10 ≤ l ≤ 10	−8 ≤ h ≤ 8, −12 ≤ k ≤ 12, −16 ≤ l ≤ 16
Reflections collected	15,163	17,559
Independent reflections	3375 [R_int_ = 0.0678, R_sigma_ = 0.0610]	3363 [R_int_ = 0.0435, R_sigma_ = 0.0340]
Data/restraints/parameters	3375/2/332	3363/0/269
Goodness-of-fit on F^2^	1.070	1.166
Final R indexes [I >= 2σ (I)]	R_1_ = 0.0407, wR_2_ = 0.0628	R_1_ = 0.0493, wR_2_ = 0.1631
Final R indexes [all data]	R_1_ = 0.0762, wR_2_ = 0.0734	R_1_ = 0.0719, wR_2_ = 0.2379
Largest diff. peak/hole/e Å^−3^	0.17/−0.16	0.40/−0.27
Flack parameter	0.2(3)	

**Table 2:** Hydrogen Bonds from crystal structure of THQ.

D	H	A	d(D-H)/Å	d(H-A)/Å	d(D-A)/Å	D-H-A/°
O1	H1	O4	0.85(4)	1.93(5)	2.769(5)	170(5)
O4	H4	O1 ^1^	0.80(4)	1.96(4)	2.755(5)	172(4)
O3	H3	O2 ^2^	1.05(8)	1.74(8)	2.781(5)	172(7)
O2	H2	O3 ^3^	1.10(8)	1.71(8)	2.791(5)	164(7)

^1^ +x, 1 − y, −1/2 + z; ^2^ 1 + x, 1 − y, 1/2 + z; ^3^ −1 + x, 1 + y, +z.

**Table 3:** RRDE efficiency slopes of various polyphenols.

BHT.	Chrysin	Eriodictyol	DHDM	Butein	Clovamide	Quercetin	Galangin
−0.16 × 10^4^ [22]	−1.10 × 10^4^ [24]	−2.20 × 10^4^ [24]	−8.0 × 10^4^ [21]	−11.2 × 10^4^ [21]	−12.0 × 10^4^ [25]	−15.5 × 10^4^ [23,24]	−19.0 × 10^4^ [26]

## Data Availability

Crystal data of TQ and THQ have been deposited at the Cambridge Structural Database (CSD) and are available at https://www.ccdc.cam.ac.uk/structures/? using Identifier CCDC numbers 2239493-2239494 (accessed on 1 January 2023).

## Supplementary Materials

The following supporting information can be downloaded at: https://www.mdpi.com/article/10.3390/antiox12030607/s1, Figure S1: Overlay of two molecules in the asymmetric unit.

## Abbreviations

TQ,	Thymoquinone;
THQ,	Thymohydroquinone;
DFT,	Density
Functional	Theory;
TBAB,	tetrabutylammonium
bromide;	RRDE,
Rotating	Ring
Disk	Electrode.
