# Supplementary figures and images for: Antioxidant Properties of Thymoquinone, Thymohydroquinone and Black Cumin (Nigella sativa L.) Seed Oil: Scavenging of Superoxide Radical Studied Using Cyclic Voltammetry, DFT and Single Crystal X-ray Diffraction
Source: Antioxidants (Basel). 2023 Mar 1;12(3):607. doi: 10.3390/antiox12030607 (PMC10045468; doi:10.3390/antiox12030607)

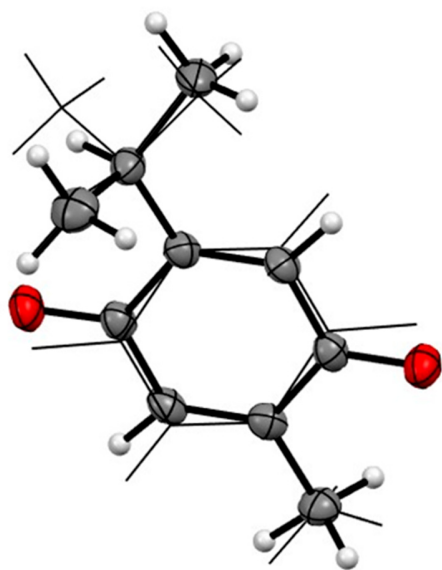

**Figure S1.** Overlay of two thymoquinone molecules in the asymmetric unit.

Supplement: Supplementary file 1 [file antioxidants-12-00607-s001.zip › antioxidants-2237939-supplementary.pdf]
